# A Radical Operation for Varicocele

**Published:** 1901-03

**Authors:** 


### A Radical Operation for Varicocele.

(Narath, Wiener. klin. Wochenschr., No. 4, 1900).—In well pronounced cases of varicocele, the writer regularly found an enlargement of the inguinal canal. He resects the main trunk of the spermatic vein in the inguinal canal, and then closes the canal. The author describes the operation, which is performed in ten or fifteen minutes. He operated on twenty-one cases in this manner with good results. After observing patients for two or three years there was no recurrence.—*Medical Fortnightly.*

